# *In vivo* Evaluation of Inflammatory Bowel Disease with the Aid of μPET and the Translocator Protein 18 kDa Radioligand [^18^F]DPA-714

**DOI:** 10.1007/s11307-014-0765-9

**Published:** 2014-07-12

**Authors:** Nicholas Bernards, Géraldine Pottier, Benoit Thézé, Frédéric Dollé, Raphael Boisgard

**Affiliations:** 1 Inserm, Unité 1023, Université Paris Sud, 91400 Orsay, France; 2 Commissariat à l’Energie Atomique et aux Energies Alternatives, Direction des Sciences du Vivant, Institut d’Imagerie Biomedicale, Service Hospitalier Frédéric Joliot, 4, place du Général Leclerc, 91400 Orsay, France

**Keywords:** Inflammation, TSPO, DPA-714, Inflammatory Bowel Diseases, PET imaging

## Abstract

**Purpose:**

The purpose of the study was to validate [^18^F]DPA-714, a translocator protein (TSPO) 18 kDa radioligand, as a probe to non-invasively quantify the inflammatory state in inflammatory bowel disease (IBD) animal models.

**Procedures:**

Quantitative positron emission tomography (PET) imaging of intestinal inflammation was conducted with 2-deoxy-2-[^18^F]fluoro-D-glucose ([^18^F]FDG) a glucose metabolism surrogate marker and [^18^F]DPA-714 a ligand of the 18 kDa TSPO, on two IBD models. The first model was induced using dextran sodium sulfate (DSS), creating global inflammation in the colon. The second model was induced by rectally administering trinitrobenzenesulfonic acid (TNBS), creating local and acute inflammation.

**Results:**

The level of inflammation was analyzed using PET imaging on days 7 and 8. The analysis obtained with [^18^F]DPA-714, yielded a significant difference between the DSS treated (0.50 ± 0.17%ID/cc) and non-treated rats (0.35 ± 0.15%ID/cc). [^18^F]FDG on the other hand did not yield a significant difference. We did observe a mean glucose consumption in the colon increase from 0.40 ± 0.11 %ID/cc to 0.54 ± 0.17 %ID/cc. In the TNBS model, the uptake level of [^18^ F]DPA-714 increased significantly from 0.46 ± 0.23%ID/cc for the non-treated group, to 1.30 ± 0.62%ID/cc for those treated. PET signal was correlated with increased TSPO expression at cellular level.

**Conclusions:**

Results indicate that [^18^F]DPA-714 is suitable for studying inflammation in IBD models. [^18^F]DPA-714 could be a good molecular probe to non-invasively evaluate the level and localization of inflammation. Moreover, *in vivo* imaging using this TSPO ligand is potentially a powerful tool to stage and certainly to follow the evolution and therapeutic efficiency at molecular level within this disease family.

## Introduction

Inflammatory bowel disease (IBD) is a disease family that has not been getting the attention that it warrants. The incidence has been slowly rising throughout much of the industrialized world, and is not limited to adults, but also affects children [1].

IBD is comprised of multiple diseases, including Crohn’s disease (CD) and ulcerative colitis (UC), which all have aspects of inflammatory responses, ranging from acute to chronic stages, and in many cases include both. The whole gastrointestinal tract can be affected from CD while UC is limited to the cecum, colon, and rectum [2]. Precise localization of the inflamed sites is one criteria permitting differential diagnosis between different types of diseases.

The fact that this disease family can have such large variations, and yet present itself with similar if not identical symptoms, makes it very difficult to diagnose and to differentiate. The origins of the diseases are unknown, but there seems to be a tendency for the disease to be more prevalent in the Western world, and in particular, within urban areas; this is not to say that the levels or the incidence outside of the urban Western world are lower, but rather that the methods of detection and definition of what constitutes IBD are similar in these areas [3]. There may be multiple factors which contribute to the development of the diseases ranging from genetic pre-dispositions to lifestyle and stress exposure [4].

Inflammation is only the beginning of medical concerns associated with IBD. If IBD is left untreated, it can lead to cancer of the intestine. Even if this does not happen, there is still a detrimental impact on daily lifestyle. It is not completely clear how to avoid suffering from IBD, but early detection would allow early treatment and thus represent a way by which suffering could be minimized. Moreover, the disease progression and evaluation of the therapeutic efficiency also lack pertinent criteria.

Currently, the clinical evaluation consists of various exams and exam types ranging from physical symptoms, such as bloody diarrhea (a cardinal symptom), to serology, endoscopic exploration, radiological analysis, and nuclear medical investigations [5]. Preclinical investigations oftentimes use rodents which have had IBD symptoms induced by various means. The general assessment of IBD *in vivo* is commonly carried out by observing bloody diarrhea and by scoring body weight changes. *Post mortem* analyses include measurements of the colon length, cytokine and chemokine concentrations along with histological scoring [6]. These last years, only few articles illustrate the interest of *in vivo* imaging using a non-specific radiotracer, which is based on the increase of glucose metabolism, for the evaluation of IBD in both human and preclinical models [7, 8].

*In vivo* molecular imaging represents an attractive means to characterize these types of pathologies, especially as it is non-invasive. Of these methods, positron emission tomography (PET) is a powerful technique allowing the clinician to access molecular information *in vivo*. PET imaging has been present in clinical applications since the 1970s and is now routinely used for diagnosis, prognosis, and theranostics. Due to the fact that it is a quantitative, specific, and sensitive imaging modality, this non-invasive *in vivo* technique is well adapted for early detection of various diseases. PET is, however, based on the use of dedicated radiotracers, the most well-known being 2-deoxy-2-[^18^F]fluoro-D-glucose ([^18^F]FDG), a surrogate marker for glucose metabolism. Recently, Lapp, but also Shyn and Ostuni and collaborators have shown that PET could indeed be used for the detection of an IBD related disease with the use of [^18^F]FDG [9–11].

[^18^F]DPA-714 is a recently developed radioligand of the translocator protein 18 kDa (TSPO) with improved signal to noise ratios as compared to previously used radiotracers and especially [^11^C]PK11195 [12, 13]. TSPO, formerly known as peripheral benzodiazepine receptor (PBR), located on the outer membrane of the mitochondria, is overexpressed in activated macrophages and microglia. This molecular event has been used widely to investigate inflammation inside the central nervous system. TSPO increase in microglia is now presented as a hallmark of brain inflammation and our laboratories have been instrumental in the characterization and validation of this radiotracer in various preclinical models of neuroinflammation [14, 15]. However, when looking at peripheral activation of macrophages and links to the inflammatory processes, little data are currently available. TSPO expression was recently described in tissue samples of human colon suffering from IBD (CD and UC) [11]. Additionally, expression has also been characterized in a rat model of IBD using autoradiography and immunohistochemistry [11].

The use of the TSPO ligand [^18^F]DPA-714 has opened doors to exploration into inflammation within the periphery, and thus also into the domain of inflammatory bowel diseases. It allows the quantification of the inflammation found in a given area within the bowel. To confirm this capability, we implemented two animal models of IBD, one using dextran sodium sulfate (DSS) inducing high diffuse inflammation in the colon, and the other using trinitrobenzenesulfonic acid solution (TNBS) inducing inflammation mainly restricted in the area of instillation [16, 17], and demonstrated the potential of [^18^F]DPA-714 to image IBD with PET.

## Materials and Methods

### Animal Models

The animals chosen were male Wistar rats (Centre d’Elevage René Janvier, France) with a weight of 250 g ± 20 g. The subjects were kept in an adapted environment in which the temperature and humidity are regulated. The lights are on a 12-h cycle on and then off. The experiments were carried out in accordance with French guidelines.

Two different animal models of acute bowel inflammation were developed and implemented in the study. The first being a DSS (Sigma-Aldrich, France; MW40,000) induced model, which leads to haematochezia, and also to weight loss along with a shortening of the intestine among other consequences associated with a colitis rat model [16]. DSS induces *in vivo* intestinal inflammation and is simple, affordable, and also has a high degree of uniformity and reproducibility of most lesions in the distal colon. In this model, we added DSS at 5 % (*v*:*v*) into the drinking water of the animals from which they had *ad libitum* access throughout the duration of the experiments. [^18^F]FDG images were acquired after 7 days of treatment whereas [^18^F]DPA-714 images were acquired after 8 days of treatment. The treatment has been used successfully with rats in previous tests [18]. Non-treated rats were given plain water in their bottles and all the animals were weighed daily, and their feces state was also observed on a daily schedule. The DSS solution was exchanged on days 3 and 5 in the treated group and the non-treated group also had their water exchanged for a new bottle containing plain water at the same time points.

The second model was chemically induced by instilling trinitrobenzenesulfonic acid (TNBS) (Sigma-Aldrich) as described by the group of Seibel *et al.* [19]. Some slight alterations were made in order to adapt this model to the PET modality. Here, inflammation was induced into male Wistar rats by administering 100 μl of 50 % ethanol-water solution (to help bypass the mucosal layer of the intestine) containing TNBS (100 g/l, corresponding to 40 mg/kg body weight) 4 cm into the rectum from the anus [17]. During this administration, the rats were under anesthesia using 2.5 % isoflurane in 100 % O_2_. This anesthesia was maintained for an extra 10 min post instillation in order to reduce displacement and possible leakage of the chemical. Non-treated animals were injected with 100 μl of ethanol and water (1/1, *v*:*v*).

The animals were then placed into individual cages and their weights were measured on a daily basis and their feces state was recorded. Inflammation was evaluated with PET imaging on day 7 using [^18^F]FDG and on day 8 using [^18^F]DPA-714 after TNBS administration.

### PET Radiotracer

[^18^F]FDG was purchased from the commercially available source Cyclopharma S.A. (Clermont-Limagne, France). [^18^F]DPA-714 was produced on site according to slight modifications of procedures already reported [20] and using a commercially available GE TRACERLab FX-FN synthesizer [21]. Ready-to-inject, >99 % radiochemical pure [^18^F]DPA-714 (formulated in physiological saline containing less than 10 % of ethanol) was obtained with 15–20 % non-decay-corrected yields and specific radioactivitiy level at the end of the radiosynthesis ranging from 37 to 111 GBq/μmol.

### Image Acquisition

The PET and PET/CT images were acquired using two separate machines. The first one is a Siemens INVEON PET and the second a Siemens INVEON PET/CT, both being dedicated small animal scanners. Each animal had its images acquired in the same machine each day. The animals were kept normothermic with the help of a heating carpet. Rats were anesthetized using 3 % isoflurane in pure oxygen and kept sedated throughout the acquisition. For [^18^F]DPA-714, images were acquired dynamically during a time span of 60 min, beginning at the same time as the injection. Each animal was injected with 37 ± 2 MBq (1.0 mCi ± 0.054 mCi). The [^18^F]FDG acquisitions (30 min) were sorted into three frames of 10 min each and the [^18^F]DPA-714 images were sorted into 16 frames. Frames 1–5 each lasted 1 min, frames 6–10 lasted 2 min, frames 11–13 each lasted 5 min, and the remaining frames (14–16) lasted for 10 min each. The energy discrimination was set at 350 and 650 KeV. The list-mode acquisition was as described previously and the data files were histogrammed into three-dimensional sinograms with a maximum difference of 47 and a span of 3 [20].

The attenuation correction of the Siemens INVEON PET was measured with the help of a Cobalt-57 point source. The Siemens INVEON PET/CT carried out the attenuation correction measurement with the use of its CT imaging capabilities and settings, which are implemented directly by the manufacturer. In order to obtain the best resolution possible within the intestines, we implemented a Fourier rebinning and an OSEM 2D reconstruction method.

### Image Analysis

The reconstructions of the PET images were carried out using ASIPro VM (CTI Concorde Microsystem Analysis Tools and System Setup/Diagnostic Tool). The image analysis and quantification of the radioactivity uptake in the regions and volumes of interest (ROI’s and VOI’s) were carried out with the use of BrainVisa/Anatomist Version 4.3.0 [22].

Delineation of the ROI’s and VOI’s was selected by means of manual segmentation and always carried out by the same user. The selected region of the intestine was 5 × 3 × 2 voxels, or a 3.6-mm^3^ volume placed on the intestinal wall.

### Gamma Counting

The ascending colon was excised once the subject had been euthanized *via* an *i.v*. injection of pentobarbital and divided into three sections. Each sample was weighed before being placed into individual tubes, which were then inserted into a Perkin Elmer Cobra II Auto-Gamma Gamma-ray counter. The results were decay-corrected and standardized for the percent injected dose, sample weight, and the body weight of each animal, which corresponds to the standardized uptake value (SUV).

### Immunohistochemistry and Morphological Analysis

*Post mortem* tissue samples were excised, once the [^18^F]DPA-714 images had been acquired, and fixed with 4 % paraformaldehyde (PFA, Sigma-Aldrich) in Phosphate-buffered saline (PBS, pH 7.4 Sigma-Aldrich, France) for 2 h followed by cryopreservation by incubation in PBS, pH 7.4 containing 20 % sucrose for 24 h. The intestines were then cut into thirds (ascending, transverse, and descending sections), immersed in embedding tissue medium (Shandon M-1 Embedding Matrix, Thermo, USA), and frozen rapidly in liquid nitrogen. Immunohistochemistry (IHC) was then carried out on slices of 5 μm thickness taken from the specimen. Anti-TSPO marker (NP-155, a kind gift of Dr. Makoto Higuchi, The National Institute of Radiological Sciences, Chiba, Japan) was used on individual slices.

Non-specific binding were blocked with 5 % BSA and 0.5 % Tween 20 in PBS (5 min, room temperature (RT)) and incubated (1 h, RT) with primary antibodies as follows: Rabbit anti-TSPO antibody (NP155, 1:500) diluted in 5 % BSA and 0.5 % Tween 20 in PBS, and, after PBS washes (three times), sections were incubated (30 min, RT) with Alexa Fluor-488 goat anti-rabbit (A11034, 1:1000; Invitrogen, France), diluted in 5 % BSA and 0.5 % Tween 20 in PBS.

### Hematoxylin and Eosin Staining

The frozen slides containing slices of the intestines were placed into fresh PBS for rehydration 5 min before being placed into deionized water for 1 min. The slides were then introduced for 5 min into a Hematoxylin solution ready to use (Sigma-Aldrich, France). Once this was carried out, the slides were placed into tap water for 5 min before being placed into a series of 5-min-baths of deionized water, followed by Eosin-Y solution (LABOnord, Templemars, France), 90 % ethanol in water, 100 % ethanol, and finally toluene for 15 min. Once this was done, the slides were individually mounted with the Eukitt quick-hardening mounting medium (Sigma-Aldrich, France), by placing a few drops onto the slide and then laying a cover slip over them.

## Results

**Fig. 1.**
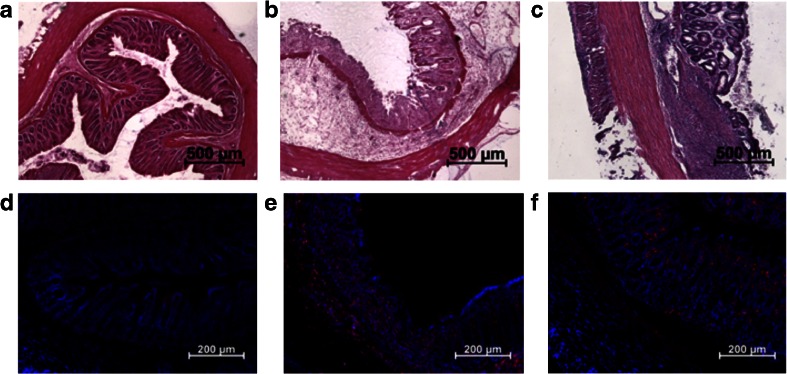
H&E staining and immunohistochemical observations using TSPO antibody (NP155, red, DAPI in blue). In non-treated rat: **a** H&E staining and **d** NP155; in rat receiving DSS in water during 7 days: **b** H&E staining and **e** NP155; and, in TNBS instilled rats: **c** H&E staining and **f** NP155.

As illustrated by the group of Lacapere, there is an overexpression of TSPO in the rat colon of the DSS-induced IBD animal model after 7 days of treatment [11]. To evaluate the possibility to both visualize and quantify the expression of TSPO in IBD, we reproduced the inflammatory model in our laboratories. In the DSS rat model, large alterations to the structure of the colon can be observed when compared with samples of non-treated animals, as illustrated in Fig 1. Microscopic observations on the different sections of the colon (ascending, transverse, and descending) of treated animals revealed erosion along the entire colon. This alteration was also associated with rectal bleeding and a loss of weight in treated animals (data not shown). The non-treated animals showed no such signs and have intact crypts as illustrated on Fig. 1a. TSPO expression was investigated on adjacent tissue samples. Representative images (Fig. 1e, f) clearly show the increased presence of TSPO in the colon of treated animals with high infiltration in the eroded *lamina propria* and high density at the base of the intestinal wall (*lamina muscularis/mucosae*). In comparison to non-treated animals, TSPO positive cells were mainly found at a lower density within the *lamina propria* (Fig. 1d).

In the TNBS model, due to the direct local administration of the inflammatory agent, the phenotype was different with the inflammation localized mainly in the upper part of the ascending colon (around 4 cm from the anus), as opposed to the global inflammation found in the first (DSS) model. In this second model, the H&E staining presented in Fig. 1a, c indicates a morphological difference between a treated animal and a healthy animal, as indicated by an alteration of the microvilli architecture of the colon in the area of instillation. TSPO staining revealed an accumulation of TSPO positive cells at the base (*lamina mucosae*) and at the extremity of the microvilli (Fig. 1f).

In order to investigate the utility of [^18^F]DPA-714 to provide information concerning the inflammatory processes *in vivo,* we performed PET imaging on non-treated and treated rats and compared the uptake of this TSPO radioligand with [^18^F]FDG, a radiotracer used previously to visualize IBD in humans. A PET imaging session was carried out on day 7 post treatment induction, using [^18^F]FDG, and on day 8 with [^18^F]DPA-714 on the same animals.

**Fig. 2.**
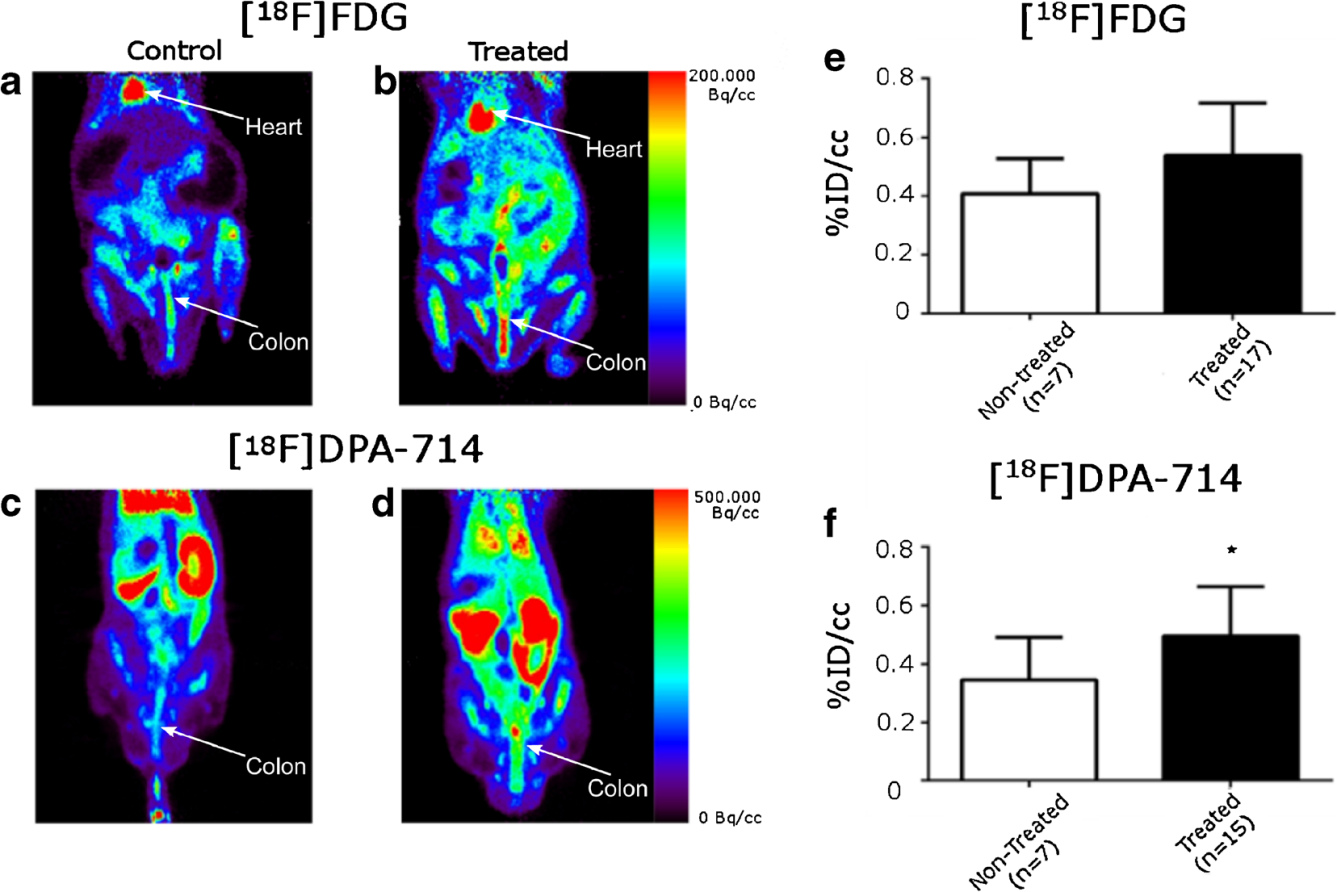
Whole body PET images on control and DSS treated animals. [^18^F]FDG in **a** control and **b** treated animals; [^18^F]DPA-714 in **c** control and **d** treated animals. *Color scales* are different ranging from 0 to 200 MBq/cc for [^18^F]FDG and to 500 MBq/cc for [^18^F]DPA-714 images. Quantification of **e** [^18^F]FDG and **f** [^18^F]DPA-714 measured on PET images in area with highest uptake within the colon on control and DSS treated rats.

Figure 2 illustrates representative images of non-treated (2a, c) and DSS treated animals (2b, d) using [^18^F]FDG (2a, b) and [^18^F]DPA-714 (2c, d) at 7 and 8 days, respectively, along with the corresponding graphs (Figure 2 e, f, respectively). For both tracers, an increased uptake in the colon of treated animals in comparison to non-treated animals was observed. The regions of interest were manually drawn with identical sizes for all images on the colon wall in the area with the highest signal. Quantification of the radioactivity uptake after 60 min of acquisition revealed that for [^18^F]FDG, the mean glucose consumption in the colon increased from 0.40 ± 0.11 %ID/cc to 0.54 ± 0.17 %ID/cc (Fig. 2). This increase identified the inflammatory processes but was not sufficiently high to provide a significant difference between the two groups of rats (*P* = 0.053). Similarly, quantification of [^18^F]DPA-714 in the colon of control and treated animals revealed a significant increase in uptake level from 0.35 ± 0.15 %ID/cc to 0.50 ± 0.17 %ID/cc (*P* = 0.040, Fig. 2). This increased significance correlates to the overexpression of TSPO in colon tissue samples as illustrated on the Fig. 1e.

**Fig. 3.**
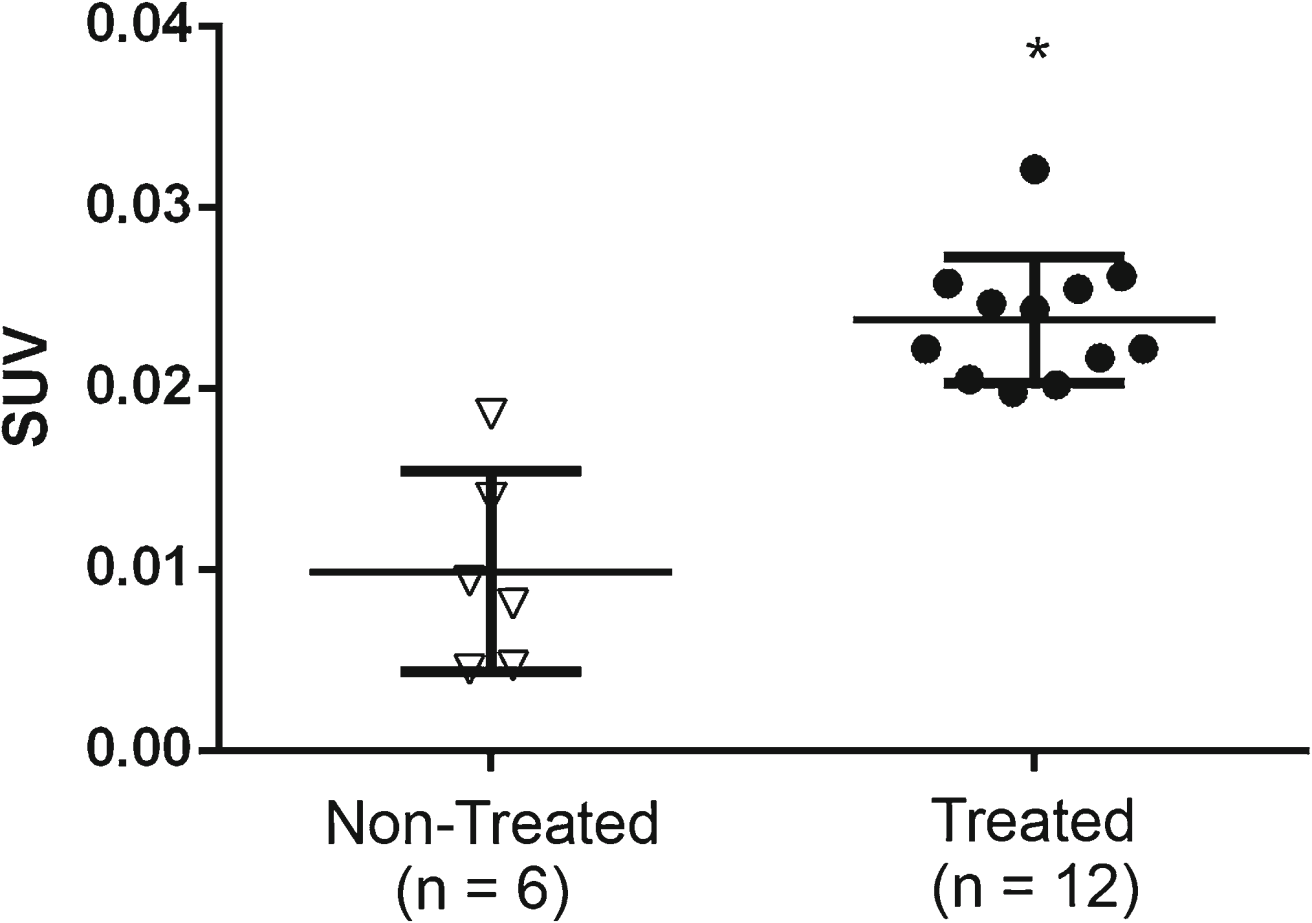
Gamma counting of the excised sections of intestines revealed a difference of [^18^F]DPA-714 presence between the two groups. (* = *p* value < 0.0001) Results are expressed in SUV.

In Fig. 3, one can observe that the PET images and the subsequent analysis, were verified/validated with the gamma counting. Here, we also observed a significant difference between the treated and control groups where *p* < 0.0001.

**Fig. 4.**
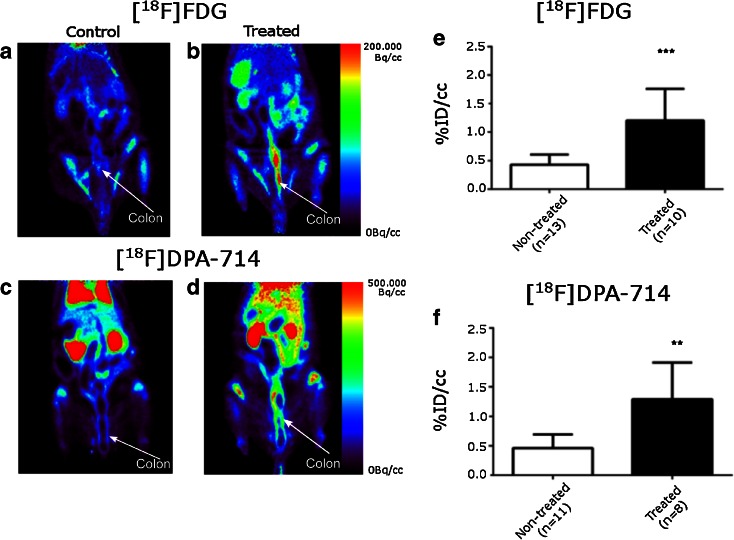
Whole body PET images on control and TNBS treated animals using [^18^F]FDG in **a** control and **b** treated animals; [^18^F]DPA-714 in **c** control and **d** treated animals. *Color scales* are different ranging from 0 to 200 MBq/cc for [^18^F]FDG and to 500 MBq/cc for [^18^F]DPA-714 images. Quantification of **e** [^18^F]FDG and **f** [^18^F]DPA-714 measured on PET images in area with highest uptake within the colon on control and TNBS treated rats. (* = *p* < 0.0006 for [^18^F]FDG and *p* < 0.0058 for [^18^F]DPA-714).

In the TNBS model, in contrast to the DSS model, the inflammation is localized mainly in the area of administration of the immunogenic stimulating agent. PET image analysis clearly illustrated that [^18^F]DPA-714 was able to highlight a difference in inflammatory status between the non-treated and the treated animals. Figure 4 illustrates the increased uptake for both tracers (images a–d and graphs e and f), [^18^F]FDG and [^18^F]DPA-714. Treated animals (Fig. 4b, d) show a more localized area of inflammation when compared to the distribution of the tracers in the DSS model (Fig. 2b, d). For both tracers, the area of increasing signal is larger than the theoretical area of instillation due to either a diffusion of the TNBS during experimental procedure or to a propagation of inflammation to a large portion of this organ.

For both [^18^F]FDG and [^18^F]DPA-714, the inflammation induced a significant increase of tracer accumulation from 0.43 ± 0.18 to 1.20 ± 0.56 %ID/cc for [^18^F]FDG and 0.46 ± 0.23 to 1.30 ± 0.62 %ID/cc for [^18^F]DPA-714 (*p* < 0.0006 for [^18^F]FDG and *p* < 0.0058 for [^18^F]DPA-714, Fig. 4).

**Fig. 5.**
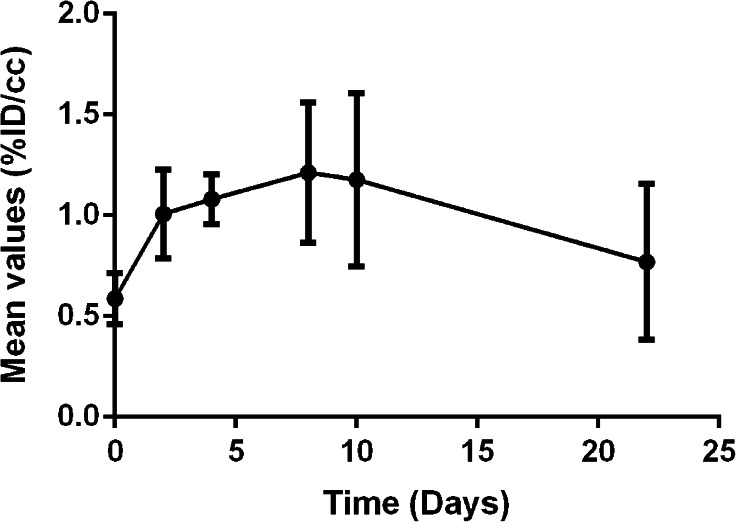
Characterization of the TSPO expression over time in a rat model of local inflammation within the colon (TNBS rat model).

The objective of molecular *in vivo* imaging is to visualize the process of interest, in our case inflammation in an IBD model, but mainly to enable analysis and quantification of a dynamic process. In order to evaluate the potential of [^18^F]DPA-714 to achieve this goal, we followed the inflammation over a period of 22 days in a group of six animals. The first images were acquired on each rat 1 day before the instillation of the TNBS to measure the basal value of [^18^F]DPA-714 uptake in the middle of the colon for each rat. After induction, the animals were imaged at different days (1, 2, 4, 8, 10, and 22) as represented in Fig. 5. This figure indicates an increase of [^18^F]DPA-714 uptake until day 8/10 after which the mean signal decreased slowly to reach basal value at 22 days post induction. A large standard deviation was observed for the mean values at days 8, 10, and 22, illustrating the highly variable inter-individual evolution of the disease.

## Discussion

IBD is a serious health issue, which appears to be increasing throughout the Western world. It is a disease which does not discriminate between ages, although it does tend to be present in higher numbers among the weaker of society, namely the young and aged groups. IBD, as a family of diseases, including Crohn’s disease and UC, can in many instances turn chronic, at which point there is no treatment for the disease itself, only its symptoms. This in itself will degrade the quality of life and result in additional medical expenses, which could possibly be avoided by early detection or improved diagnostics to evaluate the inflammatory component of the disease. PET imaging, using an appropriate radiotracer, offers this opportunity. It allows the researcher/medical doctor to observe a difference at a molecular level, which could aid in early diagnosis. This diagnosis, however, is only as good as the tools that are used. Thus far, the tools have not been focused on a specific aspect of inflammation, but rather on a symptom of the inflammation. Thus, they currently lack specific techniques for imaging and detection of IBD. We are now capable of producing improved tools and using them more effectively. In this study, we investigated two IBD models reflecting both local and diffuse inflammation of the digestive tract using PET imaging. We demonstrate that with the use of an adapted tracer [^18^F]DPA-714, we are able to detect, visualize, and quantify a difference between the non-treated animals and those that underwent the treatments in order to induce IBD symptoms (as seen in Fig. 2 and 4).

The interest of this study was multifaceted. First, we wanted to establish two different (reproducible) models of inflammatory bowel disease, with different origins. Second, we wanted to prove the relationship between tissular expression of TSPO and the specific uptake of [^18^F]DPA-714 obtained *via* molecular imaging. Third, to show that imaging can provide a quantifiable difference between sick and healthy subjects. Fourth and foremost, we wanted to validate [^18^F]DPA-714 as a probe for peripheral inflammation, which has to date been used mainly for neuroinflammation.

In addition to this list of objectives, we examined another aspect of molecular imaging, specifically for PET, which allows the user to follow the advancement and characterize of a disease or any other molecular process of choice. This approach was illustrated by the dynamic description of the inflammatory status using [^18^F]DPA-714 in the TNBS model (Fig. 5). Following the uptake of the radioligand in the instillation area, we were able to demonstrate the ability to detect varying levels of inflammation of the disease for each individual animal.

Our first objective had been to reproduce the models used by the groups of Dr. Ostuni and Dr Seibel [11, 19]. This was accomplished and used to detect inflammation in each induced animal and subsequently verified with HE staining, and also by labeling with Iba1 (macrophages) and NP-155 (TSPO 18kDa). In each case, we observed a marked structural difference between the two groups as well as an increase in signal on the treated animals from the markers. The differences can be seen in images on Fig. 1d–f.

This invasive evaluation of inflammatory statue (using biopsies) can certainly be characterized using PET as a quantifiable imaging tool. Side effects of these intestinal treatments (diarrhea and reduced gut motility) were found in both DSS and TNBS models, respectively. These two symptoms have a drastic impact on the size of the gut, the first one reducing the thickness making it difficult to visualize the intestine wall (often empty with a collapsed lumen) and the second one with an increase of the diameter due to the accumulation of feces. In the case of preclinical imaging on small animals, the access to conventional CT anatomical reference images is not possible due to the lack of soft tissue contrast. The other limit in small animal imaging is the spatial resolution of the PET camera in regard to the thickness of the intestinal wall in rodents. This resolution, coupled with the size of the structure impacts the quantitative approach due to a high partial volume effect artificially decreasing the uptake value observed in comparison to the result expected in larger animal model or in humans. To accurately compare data among different animals, with different pathological models and different tracers, we choose to define a small volume (5 × 3 × 2 voxels) that we placed on the areas of highest uptake of each animal, and then compared this volume between the groups.

Once we quantified the images, we found a difference between the results of the two tracers on the same group. The results on Figs. 2 and 5 revealed different levels of signal when comparing the healthy groups to each other. This means that the healthy group of the DSS [^18^F]FDG animals yielded slightly different results than the same group did with the [^18^F]DPA-714 radiotracer. This is understandable and explainable when looking at what each tracer is actually targeting. [^18^F]FDG is a glucose analog which indicates an increase of glucose consumption, which in itself can be indicative of other on-going processes such as cancer, cell regeneration, or muscle contraction as seen in peristalsis [23, 24]. Fibroblasts also have an increase of glucose consumption when they are exposed to inflammatory mediators such as tumor necrosis factors (TNF) [25]. This tracer has been described as a good marker of inflammatory processes but it reflects global impact on different cell populations and biological processes.

On the other hand, [^18^F]DPA-714 has not yet been shown to be increased in different cell populations except in immune white cells. This TSPO ligand has been presented as a tracer with high affinity and good specificity. Image quantification of [^18^F]DPA-714 uptake correlated with the amount of TSPO, as published previously and provides indirect information about white blood cell activation in the tissue of interest [15, 26]. In the TNBS model, the uptake at 7 days of [^18^F]FDG and on day 8 of [^18^F]DPA-714 is higher than in DSS model (Fig. 4). These differences illustrate a higher level of local inflammation in this second model.

Another difficulty we faced in one of the preclinical models was the localization of induced inflammation. Here, in the second model, we had to choose a distance at which the TNBS should be induced. We had to avoid the upper digestive tract as this is the region in which the [^18^F]DPA-714 is eliminated by, thus inhibiting us from distinguishing the potential region of inflammation and the regular elimination. Inducing the TNBS too close to the anus on the other hand was also disadvantageous as the bladder is located in the vicinity. This poses a problem as it is the renal pathway by which [^18^F]FDG is eliminated, also obstructing the image quantification. Due to these constraints, we decided to introduce the syringe at a depth of 4 cm from the anus. In larger animals or human, these problems would not be significant due to the fact that in human, [^18^F]DPA-714 is also largely eliminated by the hepatic pathway but with high gall bladder accumulation limiting the radioactivity signal in the intestine. In rats, the lack of gall bladder prevents molecular imaging of the upper part of the intestine.

Beyond this, we used [^18^F]DPA-714 to determine the point at which the level of TSPO was at its highest in the TNBS model in order to characterize the dynamic inflammatory processes in this model. This was done by acquiring images at various time points post TNBS induction, illustrating the possibility to repeat image acquisitions and the possibility to follow the evolution of the disease in an individual patient. While the data revealed the time point at which one can observe the highest level of inflammation, one can also observe heterogeneity, post peak, which revealed a large standard deviation. The origin of the heterogeneity is most likely linked to the disease severity. However, a direct correlation between the uptake of [^18^F]DPA-714 and a clinical score of the disease was not possible in this study due to the limited number of animals used for the dynamic evaluation (*n* = 6). This proof of principle can of course be extrapolated to humans with a protocol dedicated to the evaluation of therapeutic efficiency or disease progression.

## Conclusion

In this study, we report for the first time, the ability to distinguish a difference between IBD affected and healthy animals using PET imaging and gamma counting with the use of the TSPO radioligand [^18^F]DPA-714 in two separate models. [^18^F]FDG also provided a positive contrast in the intestine of sick animals. However, the statistical significance was less than that of the [^18^F]DPA-714. Beyond this, we also demonstrated the ability to follow a disease evolution over time with the use of [^18^F]DPA-714. Studies such as these demonstrate the potential applications of this TSPO ligand [^18^F]DPA-714 which may improve the clinical diagnosis for inflammatory bowel disease patients.
